# Training and transfer effects of extensive task-switching training in students

**DOI:** 10.1007/s00426-018-1059-7

**Published:** 2018-07-17

**Authors:** Xin Zhao, Haien Wang, Joseph H. R. Maes

**Affiliations:** 1 Behavior Rehabilitation Training Research Institution, School of Psychology, Northwest Normal University, 967 East Anning Road, Lanzhou, 730070 China; 2 Donders Institute for Brain, Cognition and Behaviour, Centre for Cognition, Radboud University, PO. Box 9104, Nijmegen, 6500 HE The Netherlands

## Abstract

The capacity to switch between tasks is a central component of executive functioning. Previous studies assessing effects of task-switch training have revealed mixed results, both in terms of processes that may be improved and the extent of beneficial effects on non-trained tasks. These studies primarily used few training sessions, which may have limited training and transfer effects. Here, 31 students were trained for 21 days on a cued switching task. Both the trained group and an active control group (*n* = 29) performed a number of cognitive tasks before and after training. Training reduced both switch and mixing costs, which mostly reached an asymptote after approximately four to six training sessions, although there were residual costs at the end of training. The switch cost reduction was restricted to trials with a short cue–stimulus onset interval (CSI). Training benefitted performance on another switching task, reflecting near transfer. However, this benefit was limited to the switch cost and to trials with a short CSI. There were no beneficial effects on far-transfer tasks measuring interference control, response inhibition, working memory, and general IQ. The results suggest that the present extensive training protocol, implicating overtraining, specifically enhanced the efficiency of processes involved in preparing for the relevant upcoming task set and/or inhibition of the previous task set. However, the lack of beneficial far-transfer effects is in line with previous cognitive training studies employing fewer training sessions, suggesting that the extent of training is not critical for (not) finding transfer effects.

**Electronic supplementary material:**

The online version of this article (10.1007/s00426-018-1059-7) contains supplementary material, which is available to authorized users.

## Introduction

The ability to rapidly switch between two or more cognitive tasks is one of the core elements of executive functioning (EF; Miyake et al., 2000). This ability may involve other aspects of EF, such as inhibition, interference control, and working memory (e.g., Koch, Gade, Schuch, & Phillipp, 2010), and enables a flexible adaptation to changing environmental demands. Moreover, it is associated with healthy functioning in many daily life domains (e.g., Colé, Duncan, & Blaye, 2014; Kray & Lindenberger, 2000; Yeniad, Malda, Mesman, van Ijzendoorn, & Pieper, 2013; Vaughan & Giovanello, 2010). For these reasons, the question of the trainability of task-switching ability in healthy and clinical populations has received considerable attention (e.g., Grönholm-Nyman et al., 2017; Karbach & Kray, 2009; Minear, & Shah, 2008; Pereg, Shahar, & Meiran, 2013; Zinke, Einert, Pfenning, & Kliegel, 2012). However, this attention is still relatively little when compared to the body of literature on the success of trainings directed at another component of EF, namely working memory (WM; e.g., see Melby-Lervåg, Redick, & Hulme, 2016, Soveri, Antfolk, Karlsson, Salo, & Laine, 2017, for recent reviews).

Regarding the effect of task-switching training, one can make a distinction between two aspects. The first concerns effects on performance parameters related to the trained task itself. The second concerns training benefits for non-trained tasks that are either structurally closely related to the trained task, also known as near-transfer effects, or not (far-transfer effects).

Training-task performance parameters involve the response time (RT) or accuracy switch and mixing costs (e.g., Kiesel et al., 2010). The switch cost refers to the difference in RT or accuracy on trials from mixed-task blocks involving a task switch and those involving a repetition of the previous task, with the former generally yielding longer RTs and more errors than the latter. The mixing cost signifies the RT or accuracy difference between non-switch trials from mixed-task trial blocks and trials from single-task blocks, with the former generally yielding longer RTs and more errors than the latter. The switch cost is assumed to result from costs associated with reconfiguration of the task set (Rogers & Monsell, 1995), overcoming interference effects between the previous and current task execution (Allport, Styles, & Hsieh, 1994), or both (Monsell, 2003; Kiesel et al., 2010). The mixing cost is suggested to result from resolving task-set conflicts or stimulus ambiguity during mixed-task trials (Rubin & Meiran, 2005), or from differences in arousal level or working memory load between single-task and mixed-task blocks (Rogers & Monsell, 1995).

In general, research suggests that practicing task switching reduces the switch cost (e.g., Berryhill, & Hughes, 2009; Karbach & Kray, 2009; Kray & Fehér, 2017; Kray, Karbach, Haenig, & Freitag, 2012; White & Shah, 2006; Zinke et al., 2012), and the mixing cost (e.g., Minear, & Shah, 2008; Soveri, Waris, & Laine, 2013; Strobach, Liepelt, Schubert, & Kiesel, 2012). However, at least for the switch cost in tasks using a separate cue to indicate the to-be-performed task, practice-induced reductions may be limited to trials with a short cue-to-stimulus interval (CSI; Meiran, Chorev, & Sapir, 2000; Wendt, Klein, & Strobach, 2017). Such trials, relative to those with a long CSI, induce a large switch cost to begin with before training and training may specifically enhance the efficiency of processes involved in task preparation and/or reducing interference from the irrelevant task set.

The outcome of studies examining training-induced transfer effects is somewhat mixed. Most studies found beneficial near-transfer effects in the form of reduced switch and/or mixing costs for untrained switching tasks (e.g., Anguera et al., 2013; Karback & Kray, 2009; Kray & Fehér, 2017; Kray et al., 2012; Minear & Shah, 2008; Pereg et al., 2013; White & Shah, 2006; Zinke et al., 2012). However, the results concerning far transfer are less conclusive. Whereas some studies found beneficial transfer to tasks measuring other aspects of EF, such as inhibition and WM (Anguerra et al., 2013; Karbach & Kray, 2009; Kray et al., 2012), other studies found no, or much more limited far transfer (e.g., Dörrenbächer, Müller, Tröger, & Kray, 2014; Kray & Fehér, 2017; Pereg et al., 2013; Soveri et al., 2013; von Bastian & Oberauer, 2013; Zinke et al., 2012). These studies differ in a number of aspects that each could have affected the magnitude of far-transfer effects, such as the age of the participants (children, adolescents, young adults, older adults), the type of the participants (clinical vs. non-clinical), the nature of the training task (e.g., in game setting or not, cued or un-cued switching task), and the duration of the training protocol (ranging from three to six sessions). However, so far none of these potential modulators can be identified as being consistently associated with the presence or absence of significant far-transfer effects.

Given the inconsistencies, the present study sought to further examine the effect of task-switching training on parameters of training-task performance and on near and far transfer in healthy young adults. Our study involved a combination of the following (partly) unique features. The first feature was of primary importance and concerned the use of a training protocol that was much more extensive than that used in most previous task-switching research. Specifically, we employed a large number of training sessions (21). Based on previous studies (e.g., Wendt et al., 2017), we expected the reduction of both switch and mixing costs to reach an asymptote after a relatively limited amount of training sessions but we were specifically interested in the effects that continued training might have on transfer effects. The general literature on cognitive and motor skill learning suggests that extensive amounts of overtraining may be necessary to make a skill fully automatic, to enhance retention of that skill over time, and to induce changes in brain activation that are reflective of this automaticity (e.g, Driskell, Willis, & Copper, 1992; Kluge, Sauer, Schüler, & Burkolter, 2009; Puttemans, Wenderoth, & Swinnen, 2005). Concerning the issue of training-induced structural or functional brain changes, previous studies on the effects of working memory training suggest that extensive training is necessary to induce such changes (e.g., Jausovec & Jausovec, 2012; Olesen, Westerberg, & Klingberg, 2004; Westerberg & Klingberg, 2007) and it may well be that this also holds for other types of cognitive training, such as task-switching training. The present study is the first to assess transfer effects of extended task-switching training and the question of major interest was whether such training would yield more reliable transfer than that observed in previous research using less extensive training.

Second, we performed our extensive training while manipulating the CSI in different blocks of the training task. This was done to confirm that training specifically affects processes assumed to be responsible for switching in cued tasks, namely task-set preparation and/or interference effects. If extensive training results in more efficient task-set preparation and/or resolving of interference, training effects should specifically be present for trials with a relatively short CSI, as has been shown recently in a previous study using a more limited training (Wendt et al., 2017). Only for these trials should there be a large switch cost at the outset of training (on trials with a long CSI, participants already have enough time for preparation at the start of training), which would then be reduced in the course of training. However, despite the extensive training, we expected residual switch costs at the end of training in all CSI conditions, as frequently observed in task-switching studies (Vandierendonck et al., 2010), even after very extensive practice (Stoet & Snyder, 2007). As discussed in more detail later in Discussion section, such residual costs may originate from exogenous (target-related) processes rather than endogenous, preparation-time sensitive processes. A similar line of reasoning could be applied to the mixing cost, at least when it is assumed that this cost primarily reflects resolution of task-set conflict (Mayr, 2003; Rubin & Meiran, 2005). That is, if a bivalent target stimulus on both switch and non-switch trials of mixed-task blocks elicits a conflict between task sets (relative to what is the case on single-task trials), giving the participant more time to prepare for the next stimulus and associated task should result in a reduced mixing cost (e.g., see Lawo, Philipp, Schuch, & Koch, 2012; Rubin & Meiran, 2005, for support). The question is whether training benefits of our extensive training program in terms of a reduction of mixing cost (but not complete elimination; see Whitson et al., 2014) is also limited to conditions with relatively short CSIs.

Third, for training we used a switching task with stimuli containing two dimensions (shape and color) and that had to be categorized according to these two dimensions. Training on this task could either enhance attentional processes related to a more efficient prioritizing of attention for a specific stimulus dimension, or to more general, non-perceptual preparatory processes (see also Wendt et al., 2017). In the present study, we adopted a switching task for assessing near transfer that contained only one stimulus on each trial that could be categorized according to one of two aspects (number or parity) and also using trials with relatively long and short CSIs. Switching between these categorization dimensions did not involve switching between perceptual dimensions. The question here was whether or not we would be able to find positive transfer for this task. In case of positive transfer effects, which we expected to especially occur for short CSI trials, we would be able to conclude that training enhanced the efficiency of more abstract, non-perceptual preparatory processes rather than of stimulus-related attentional processes.

Fourth, we used a relatively large battery of tasks to assess far-transfer effects. These tasks covered important aspects of executive functioning, such as working memory, response inhibition, and interference control, in addition to a measure of fluid intelligence. The question of interest was whether our extensive task-switching training, relative to an active control group that we also used in one of our previous training studies (Zhao, Chen, & Maes, 2016), would result in beneficial effects for any of these transfer tasks.

## Method

### Participants

The study included 70 healthy undergraduate students, aged 18–23 years, from Northwest Normal University. Participants were randomly assigned to either the training or control group (see below). Six and four students were excluded from, respectively, the control and training group because of incomplete or outlying data. There was no difference in mean age of the remaining participants, *F*(1, 58) = 0.35, *p* = 0.29, or in gender distribution in the two groups, *χ*^2^(60) = 0.92, *p* = 0.34; control group: *n* = 29, 7 men, *M* = 20.14 years, SD = 0.95; training group: *n* = 31, 11 men, *M* = 19.85 years, SD = 0.85. All participants had normal or corrected-to-normal vision, were not color blind, and had no history of psychiatric or neurological disease. The students had signed an informed consent form and were paid 150 RMB for their participation.

### Pre- and post-training tests

#### Switching task

This task measured the ability to flexibly switch between two tasks (near transfer). Each trial started with the presentation of a fixation cross for 500 ms. Thereafter a cue, consisting of either a red or blue square was presented for 300, 600, or 800 ms, immediately followed by one of the digits 1–9, except 5. The cue indicated which one of two tasks was to be performed. Specifically, a red square indicated that the participant had to judge as fast as possible whether the digit was odd or even (Task A: parity judgement). In case of an odd digit, the participant had to press the letter F on a standard keyboard. If the digit was even, the participant had to press the letter J. A blue square signified that the participant had to indicate whether the digit was larger or smaller than 5 (Task B: magnitude judgement), using the letter J for < 5, and F for > 5. Hence, there were three trial types in terms of time (300, 600, or 800 ms) between the start of the cue and the start of the imperative stimulus (CSI). The digit was presented until the participant either pressed the letter F or J. The trial finished with a blank screen that was presented for 500 ms, after which the next trial started immediately. The task started with a block of 20 trials, consisting of Task A and Task B trials at different CSIs. This block was repeated until the participant responded accurately on > 80% of the trials. The practice phase was followed by three blocks of 141 trials each. During the first block, the CSI was set at 800 ms. The block started with 35 trials exclusively displaying the red cue, indicating that Task A was in effect (single-task trials). During the next 35 trials, the cue was always blue, indicating that the participant had to perform Task B (single-task trials). The next 71 trials consisted of a quasi-random mix of Task A and Task B trials (mixed trials); each task was presented 35 times (the first trial was not used for analysis) in such a way that there were 35 trials on which the current trial was the same as the previous trial (non-switch trials) and 35 trials on which the current trial was different from the preceding trial (switch trials). The second and third block of 141 trials were identical to the first block except that the CSI was set at 600 and 300 ms, respectively. The participant could have a break between trial blocks and the task lasted about 18–20 min. The dependent measures from this task were the switch and mixing costs. The switch cost is the difference in median RT on the switch and non-switch trials of the mixed-task block, based on trials with a correct response. The mix cost is the difference in median RT on the non-switch trials of the mixed-task block and the single-task trials, based on trials with a correct response. A high score represents a large switch and mixing cost, respectively. We used median rather than mean RTs to reduce the influence of outlying RTs (specifically potential extreme long RTs given the unlimited time to respond). However, analyses using mean RTs while removing outlying values resulted in the same pattern of results as those reported below.

#### Stroop color–word interference task

This task was used to assess interference control (McLeod, 1991). Each trial commenced with a 500 ms presentation of a fixation cross, followed by a blank screen that was presented for 1000 ms. Subsequently, one of four possible target stimuli was presented for maximally 1500 ms or until the participant made a response, whichever came first. The target stimulus consisted of either a congruent, incongruent, or neutral stimulus. Congruent stimuli were either a Chinese character representing the color red printed in red ink or the Chinese character representing the color green printed in green. Incongruent stimuli consisted of the character representing red printed in green, and the character representing green printed in red. Neutral stimuli consisted of the symbols ‘###’, which were either printed in red or green. The participant had to indicate the color in which the character or symbol string was printed as fast and accurately as possible by pressing F for green and J for red. A blank screen was presented after the target stimulus for a random duration between 600–1000 ms and the next trial started immediately thereafter. The task started with an 18-trial practice block that was repeated until the participant responded correctly on at least 85% of the trials. Next, the actual experiment was started, consisting of three 36-trial blocks. Twelve trials of each type (congruent, incongruent, and neutral) were randomly presented during each block. The participant could have a break between blocks and the task lasted about 10 min. The dependent measure was the difference in mean RT between incongruent and congruent trials (interference score), based on trials with a correct response and RTs > 200 ms. A high score reflects weak interference control.

#### Flanker task

This task was used to obtain an additional index of interference control (Eriksen & Eriksen, 1974). Each trial began with a 500 ms fixation cross that was followed by a blank screen, presented for a variable duration between 300–500 ms. Next, the target stimulus was shown for 1500 ms or until the participant responded, whichever came first. The next trial was initiated after a blank screen that was presented for 1000 ms. There were four different target stimuli, consisting of five arrows pointing to the right or left (congruent trials), or a central arrow pointing left that was surrounded by arrows pointing to the right (two arrows on each side), or vice versa (incongruent trials). Participants had to indicate as fast and accurately as possible the direction of the central arrow, by pressing the letter F for left and J for right on a keyboard. The main task was started after 16 practice trials, which were repeated until the participant reached an accuracy level of ≥ 85%. The main task comprised 4 blocks of 32 trials each, 16 incongruent, and 16 congruent trials, randomly presented. Participants could have a break between trial blocks and the task took about 15 min. The dependent measure was the difference in mean RT on incongruent and congruent trials, based on RTs > 200 ms and trials with a correct response. A high score reflects weak interference control.

#### Go/no-go task

A go/no-go task (Robertson, Manly, Andrade, Baddeley, & Yiend, 1997) was used to assess response inhibition. Each trial started with a fixation cross that was presented for 1000 ms. Thereafter, the target stimulus, consisting of either the letter X or Y, was presented for 600 ms, followed by a 1000 ms blank screen, implicating a response window of 1600 ms. The task began with 20 practice trials during which the participant had to respond as fast as possible to each X by pressing the letter J on a keyboard (go trials), and to refrain from responding to each Y (no-go trials). The practice phase was repeated until the participant responded correctly on ≥ 85% of the trials. The actual experiment continued with 4 blocks of 100 trials each. The participant had to respond to X and not to Y during the first two blocks. During the last two blocks, which were preceded by one or more blocks of practice trials until the participant reached an accuracy level of ≥ 85%, the participant had to respond to each Y and not to X. During each block, the letters X and Y were randomly presented and each letter was presented on 50% of the trials. The participant could have a break between trial blocks and the task lasted approximately 15 min. The main dependent measure from this test was the difference score based on the proportion of hits (correct response to go stimuli) and the proportion of false alarms (incorrect response to no-go stimuli). A high score represents a strong inhibition capacity. We also used the mean RT on go trials as an index of general vigilance or decision-making speed.

#### Numerical WM updating: easy and difficult task

Two tasks were used to measure WM ability. On each trial of the easy task, a series of single digits, from 0 to 9, were presented consecutively in the center of the screen. The length of the digit sequences, representing the different trial types, varied and consisted of 5, 7, 9, or 11 digits. Each trial type was presented an equal number of times in a random order. Participants were instructed to sequentially remember the final three presented digits. After presentation of the last digit of the sequence, a blank bar was presented on the screen and the participant had to enter the last three digits presented using the keyboard. Each trial started with a 500 ms fixation cross. Thereafter, the digits were shown for 1750 ms, followed by a blank screen that was presented for a random time between 800 and 1200 ms. The task included three trial blocks. The first block consisted of eight practice trials. The second and third blocks each contained 12 trials. The difficult WM updating task was identical to the easy task except that the digits were shown for 750 ms. Each participant completed the easy task prior to the difficult task. The dependent measure was the total number of points acquired during the task. One point was assigned for each correct digit correctly put in the correct serial position, implicating a maximum score of 72 points. This number was converted to proportion correct responses.

#### Raven’s progressive matrices test

Raven’s Advanced Progressive Matrices (RAPM; Raven, Raven, & Court, 1998) test was used as measure of nonverbal intelligence. This test consists of a series of incomplete designs. The participant is asked to select the correct part to complete the designs from eight options printed underneath. The adults completed 18 even-numbered problems from the RAPM before training and 18 odd-numbered problems during the post-training session. The maximum time allowed to work on the task was 20 min. The dependent measure was the proportion correctly solved problems.

### Computerized training program

The task used for training had the same structure as described for the pre- and post-training switching task but involved different target stimuli. Target stimuli consisted of the letters X and O, each printed in either red or green. The red square cue signaled that the participant had to indicate whether the letter was printed in red, by pressing the letter F, or green, by pressing the letter J (Task A). The blue square indicated that the participant had to indicate whether the letter was an X, by pressing the F key, or an O, by pressing the J key (Task B). We used a fixed block order, always starting with the longest CSI, to gradually increase task difficulty within each session and, in this way, to enhance the likelihood of continued motivation to work on the task. Each training session lasted about 20 min and all other details were as described for the pre- and post-training switching task.

### Procedure

All participants completed the pre-training tests on three successive days in the following order. On day 1, they performed the RAPM and Stroop tasks, on day 2, the WM and Flanker tasks, and on day 3, the go/no-go and switching tasks. The training group then completed the switching training program consisting of performing the switching task on each of 21 successive weekdays. The control group made sand paintings at the same time and in the same environment as held for the training group [see also Zhao, Chen, & Maes, (2016)]. Sand painting is practiced by a number of cultural and religious groups, such as native Americans, Buddhist monks, and Australian aborigines, and often are part of spiritual or healing ceremonies. After completion of the training or control program, all participants performed the same tests as during the pre-training assessment (post-training assessment). However, the order of presentation of the different tests now was random and two tests were performed on day 1, two tests in the morning and two tests in the afternoon, and two tests on day 2.

### Statistical analysis

The overall mean accuracy score for the training sessions and the switching, Stroop, and Flanker transfer tasks was near asymptote, which is likely also caused by the practice trials before each task that were repeated until reaching a high accuracy level (*M* = 0.96, SD = 0.04, for the training sessions, *M* = 0.94, SD = 0.07, for the switching task, *M* = 0.96, SD = 0.05, for the Stroop task, and *M* = 0.99, SD = 0.02, for the Flanker task). Moreover, ANOVA using these scores did not reveal any significant effects of major interest involving the Session (for the training) or Group (for the transfer tasks) factor, and there were no signs of reliable speed-accuracy tradeoffs (see Supplementary Material). Therefore, for the training, switching, Stroop, and Flanker tasks, we exclusively report the results of the analyses on the RT data. Two repeated measures analyses of variance (ANOVAs) were performed to evaluate the training data, with CSI (300, 600, and 800 ms) and Session (1–21) as within-subjects factors on switch cost (difference in RT between switch and non-switch trials of the mixed-task blocks) in one analysis, and mixing cost (difference in RT between non-switch trials from mixed-task blocks and trials from single-task blocks) in the other. We also performed Helmert contrasts on the training data (for the switch and mixing costs) to assess the point (session) of asymptotic responding. These contrasts determined the significance of the difference in RT on the current trial and the mean of the following trials. Consistent non-significance of this contrast from a given session onward was taken as evidence of reaching asymptotic performance on that session. Pre- and post-training-task performance was analyzed using a Group (training vs. control), and Session (pre- vs. post-training) ANOVA, using the main dependent measure(s) from each task. Significant interactions were followed up by simple main effect analyses. A Greenhouse–Geisser correction was used to correct for violation of the sphericity assumption; for the F values of the corresponding analyses, we report the degrees of freedom that result from the analysis without this correction. The alpha level was set at < 0.05 and partial eta-squared (*η*_p_^2^) was used as an effect size estimate.

## Results

### Training

**Fig. 1:**
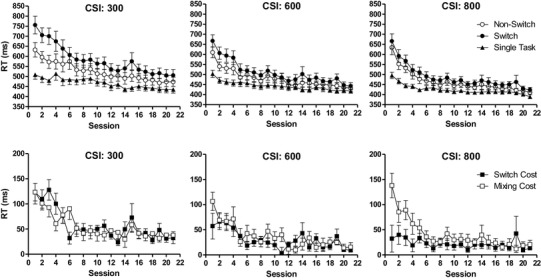
Top three panels: mean (+ SEM) of the median RT for each of the three trial types (switch, non-switch, and single-task trials) on each of the 21 training sessions. Bottom three panels: mean switch and mixing costs across training sessions. Results are shown separately for each of the three CSI conditions (300, 600, and 800 ms)

The top three panels of Fig. 1 displays the students’ mean of the median RT (+ standard error of the mean, SEM) across the training sessions for the three trial types, separated by CSI condition. The bottom three panels of Fig. 1 show the switch and mixing costs for each CSI condition across training sessions. RTs gradually decreased across the training sessions for each trial type in each CSI condition. However, for the CSI 300 and 600 ms trials, the decrease was more pronounced for switch than non-switch trials, reflecting a decreasing switching cost. For the trials in the CSI 800 ms condition, the switch cost was very small from onset on and remained relatively constant across training. Finally, the mixing cost, reflected by the RT difference between single-task and non-switch trials decreased to a more comparable extent in the three different CSI conditions than was the case for the switch cost. These impressions were statistically confirmed (see Table 1 for details of all main ANOVAs). For the switch cost, the significant CSI × Session interaction reflected a significant Session effect for the CSI 300 ms condition, *F*(20, 600) = 6.32, *p* < 0.001, *η*_p_^2^ = 0.17, and the CSI 600 ms condition, *F*(20, 60) = 2.56, *p* = 0.02, *η*_p_^2^ = 0.08, but not the CSI 800 ms condition, *F* < 1. For the mixing cost, the main effect of CSI reflected a larger mixing cost on the CSI 300 ms trials (*M* = 55.74, SD = 50.88) than on the CSI 600 ms trials (*M* = 36.19, SD = 42.91), *p* < 0.001, which did not differ from the mixing cost on CSI 800 ms trials (*M* = 40.63, SD = 49.57), *p* = 0.28. The effect of Session was also significant, but did not interact with CSI condition.

**Table 1:** Results of ANOVA on training measures, and pre- and post-training test measures for the trained and control groups

Task and measure	Main effects	Main or interaction effects	Interaction effects
Switch training			
Switch cost (RT)	CSI: *F*(2,60) = 65.79, ***p*** < **0.001**, *η*_p_^2^ = 0.69	Session: *F*(20,600) = 4.15, ***p*** = **0.002**, *η*_p_^2^ = 0.12	CSI × Session: *F*(40,1200) = 2.21, ***p*** = **0.02**, *η*_p_^2^ = 0.07
Mixing cost (RT)	CSI: *F*(2,60) = 7.61, ***p*** = **0.004**, *η*_p_^2^ = 0.20	Session: *F*(20,600) = 10.36, ***p*** < **0.001**, *η*_p_^2^ = 0.26	CSI × Session: *F*(40,1200) = 1.20, *p* = 0.29, *η*_p_^2^ = 0.04
Swc, last session	CSI: *F*(2,60) = 15.27, ***p*** < **0.001**, *η*_p_^2^ = 0.34	Trial type: *F*(1,30) = 16.29, ***p*** < **0.001**, *η*_p_^2^ = 0.35	CSI × Trial type: *F*(2,60) = 2.95, *p* = 0.08, *η*_p_^2^ = 0.09
Mic, last session	CSI: *F*(2,60) = 10.96, ***p*** = **0.002**, *η*_p_^2^ = 0.27	Trial type: *F*(1,30) = 10.80, ***p*** = **0.003**, *η*_p_^2^ = 0.27	CSI × Trial type: *F*(2,60) = 4.26, ***p*** = **0.02**, *η*_p_^2^ = 0.12
Switching			
Switch cost (RT)	Group: *F*(1,58) = 6.40, ***p*** = **0.01**, *η*_p_^2^ = 0.10	Group × CSI: *F*(2,116) = 1.32, *p* = 0.27, *η*_p_^2^ = 0.02	Group × CSI × Session: *F*(2,116) = 4.86, ***p*** = **0.02**, *η*_p_^2^ = 0.02
	CSI: *F*(2,116) = 19.06, ***p*** < **0.001**, *η*_p_^2^ = 0.25	Group × Session: *F*(1,58) = 9.44, ***p*** = **0.003**, *η*_p_^2^ = 0.14	
	Session: *F*(1,58) = 10.53, ***p*** = **0.002**, *η*_p_^2^ = 0.15	CSI × Session: *F*(2,116) = 0.91, *p* = 0.37, *η*_p_^2^ = 0.02	
Mixing cost (RT)	Group: *F*(1,58) = 1.78, *p* = 0.19, *η*_p_^2^ = 0.03	Group × CSI: *F*(2,116) = 6.49, ***p*** = **0.002**, *η*_p_^2^ = 0.10	Group × CSI × Session: *F*(2,116) = 2.73, *p* = 0.07, *η*_p_^2^ = 0.05
	CSI: *F*(2,116) = 0.95, *p* = 0.39, *η*_p_^2^ = 0.02	Group × Session: *F*(1,58) = 0.00, *p* = 0.96, *η*_p_^2^ = 0.00	
	Session: *F*(1,58) = 16.10, ***p*** < **0.001**, *η*_p_^2^ = 0.22	CSI × Session: *F*(2,116) = 1.67, *p* = 0.19, *η*_p_^2^ = 0.03	
Stroop			
Interf. score (RT)	Group: *F*(1,57) = 0.40, *p* = 0.53, *η*_p_^2^ = 0.01	Session: *F*(1,57) = 2.35, *p* = 0.13, *η*_p_^2^ = 0.04	Group × Session: *F*(1,57) = 2.40, *p* = 0.13, *η*_p_^2^ = 0.04
Flanker			
Interf. score (RT)	Group: *F*(1,58) = 0.04, *p* = 0.84, *η*_p_^2^ = 0.00	Session: *F*(1,58) = 0.13, *p* = 0.72, *η*_p_^2^ = 0.00	Group × Session: *F*(1,58) = 0.26, *p* = 0.61, *η*_p_^2^ = 0.01
WM			
Prop. correct	Group: *F*(1,58) = 0.00, *p* = 0.99, *η*_p_^2^ = 0.00	Group × Task: *F*(1,58) = 1.11, *p* = 0.30, *η*_p_^2^ = 0.02	Group × Task × Session: *F*(1,58) = 0.50, *p* = 0.48, *η*_p_^2^ = 0.01
	Task: *F*(1,58) = 12.59, ***p*** = **0.001**, *η*_p_^2^ = 0.18	Group × Session: *F*(1,58) = 0.07, *p* = 0.80, *η*_p_^2^ = 0.00	
	Session: *F*(1,58) = 16.76, ***p*** < **0.001**, *η*_p_^2^ = 0.22	Task × Session: *F*(1,58) = 1.37, *p* = 0.25, *η*_p_^2^ = 0.02	
GNG			
Hits-FA	Group: *F*(1,58) = 1.82, *p* = 0.18, *η*_p_^2^ = 0.03	Session: *F*(1,58) = 0.17, *p* = 0.69, *η*_p_^2^ = 0.00	Group × Session: *F*(1,58) = 2.62, *p* = 0.11, *η*_p_^2^ = 0.04
GoRT	Group: *F*(1,58) = 0.34, *p* = 0.56, *η*_p_^2^ = 0.01	Session: *F*(1,58) = 0.24, *p* = 0.62, *η*_p_^2^ = 0.00	Group × Session: *F*(1,58) = 10.11, ***p*** = **0.002**, *η*_p_^2^ = 0.15
RAPM			
Prop. correct	Group: *F*(1,58) = 0.72, *p* = 0.40, *η*_p_^2^ = 0.01	Session: *F*(1,58) = 0.39, *p* = 0.53, *η*_p_^2^ = 0.01	Group × Session: *F*(1,58) = 0.01, *p* = 0.93, *η*_p_^2^ = 0.00

*ps* in bold are < 0.05

*swc* switch cost, *mic* mixing cost, *interf. score* interference score

Although small in absolute terms, the RT difference between switch and non-switch trials (switch cost) was still significant on the very last training session in each CSI condition, as reflected by the outcome of a Trial Type (switch, non-switch) × CSI ANOVA (see Table 1 for details of the results). The main effect of CSI reflected a longer RT on CSI 300 ms (*M* = 489.76, SD = 142.11) than on 600 ms trials (*M* = 438.01, SD = 88.59), and on CSI 600 ms than 800 ms (*M* = 417.56, SD = 63.82) trials, *p*s < 0.02. Of primary importance, the effect of trial type was highly significant and reflected faster responding on non-switch (*M* = 439.27, SD = 88.42) than switch (*M* = 457.62, SD = 100.82) trials, and did not interact with the CSI factor. There was also evidence of a remaining RT difference between single-task and non-switch trials (mixing cost) on the very last training session. A corresponding ANOVA revealed a main effect of CSI, reflecting slower responding on CSI 300 ms (*M* = 454.49, SD = 112.61) than 600 ms trials (*M* = 425.60, SD = 68.77), *p* = 0.01, and on CSI 600 ms than 800 ms (*M* = 400.45, SD = 46.62), *p* = 0.001. The effect of Trial Type was also significant, as was the Trial Type × CSI interaction. The interaction reflected a significant trial type effect for CSI 300 ms trials (*M* = 435.55, SD = 98.94 for single-task, and *M* = 473.44, SD = 130.29 for non-switch trials, *p* < 0.001), and CSI 800 ms trials (*M* = 389.98, SD = 35.14 for single-task, and *M* = 410.92, SD = 64.37 for non-switch trials, *p* = 0.02), whereas the difference between trial types was only marginally significant for CSI 600 ms trials (*M* = 417.74, SD = 58.60 for single-task, and *M* = 433.45, SD = 85.01 for non-switch trials, *p* = 0.085).

The Helmert contrasts for the switch cost data from the CSI 300 ms condition revealed that the switch cost on each of Sessions 1–5 differed from the mean of the respective subsequent sessions, *p*s < 0.03, whereas from Session 6 on, the contrasts were non-significant, *p*s > 0.10, suggesting asymptotic responding from Session 6 onwards. For the corresponding mixing-cost data, the contrast was significant for each of the Sessions 1–6, *p*s < 0.008, except for Session 4, *p* = 0.21. From Session 7 on, the contrast was consistently non-significant, *p*s > 0.06, suggesting asymptotic responding at Session 7. For the switch cost in the CSI 600 ms condition, the contrast was significant for Sessions 2–4, *p*s < 0.05, but not for the remaining sessions, *p*s > 0.06, suggesting asymptotic responding from Session 5 onward. The pattern of results of the contrast analyses for the mixing cost during this condition was somewhat irregular. The contrast was significant for Sessions 1–3, *p*s < 0.005, but not for the remaining sessions, *p*s > 0.051, except for Sessions 9 and 13, *p*s = 0.02. Finally, for the CSI 800 ms condition, none of the contrasts was significant when looking at the switch costs, *p*s > 0.075, which corresponds with the results of the corresponding ANOVA described above (no effect of Session). Regarding mixing costs, the contrasts were significant for each of Sessions 1–5, *p*s < 0.05, but not for any of the remaining sessions, *p*s > 0.10, except for Session 9, *p* = 0.04.

### Transfer effects

**Fig. 2:**
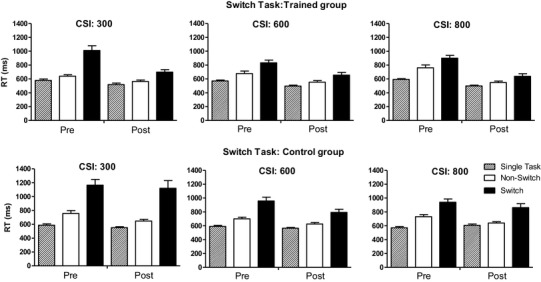
Groups’ mean (+ SEM) of the median RT on the three different trial types (switch, non-switch, and single-task trials) of the transfer switch task, separately for pre- and post-training assessment session and CSI condition

**Fig. 3:**
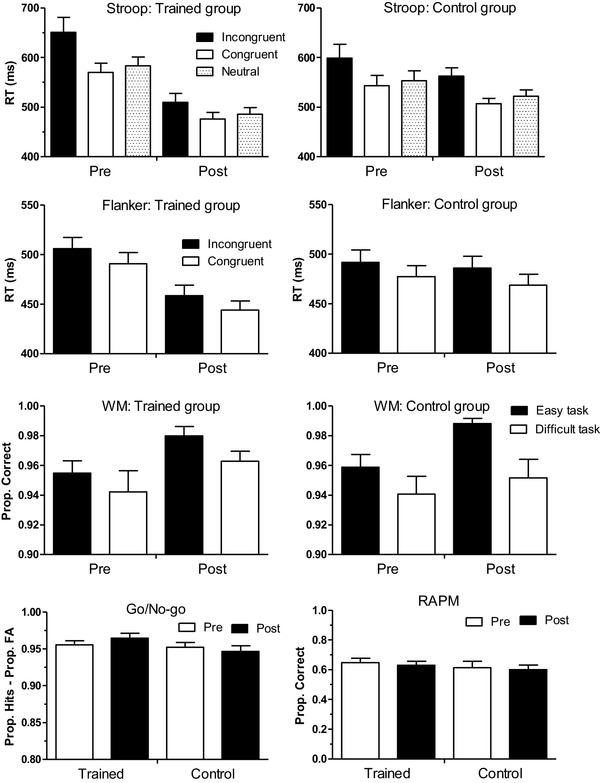
Groups’ mean (+ SEM) performance on each the five far-transfer tasks assessed during pre- and post-training sessions. Top two panels: RT for incongruent, congruent, and neutral trials of the Stroop task. Second pair of graphs from top: RT for incongruent and congruent trials of the Flanker task. Pair of graphs above bottom pair: proportion correct responses on the easy and difficult WM task. Bottom left: difference between hits and false alarms based on the go/no-go task. Bottom right: proportion correct on the RAPM task

Figure 2 shows the groups’ mean RT on the single-task, switch, and non-switch trials of the switching transfer task, separately for each CSI condition. Figure 3 displays the score on the performance measure(s) of the other transfer tasks used during the pre- and post-training sessions.

#### Switching task

Figure 2 suggests that the RT difference between switch and non-switch trials (switch cost) was smaller in the post- than pre-training session for the participants in the training group, at least in the CSI 300 ms condition. For the control group, the switch cost remained largely the same on the post- compared to pre-training session regardless of CSI condition. Regarding the RT difference between single-task and non-switch trials (mixing cost), there were no large differences between groups in any of the CSI conditions or assessment sessions. These impressions were statistically supported (see Table 1 for statistical details for this and the other transfer tasks). A Group × CSI × Session ANOVA on the switching cost revealed a significant three-way interaction, motivating a CSI × Session ANOVA for each group separately. For the trained group, this analysis revealed a significant CSI × Session interaction, *F*(2, 60) = 8.09, *p* = 0.002, *η*_*p*_^2^ = 0.21, next to main CSI and Session effects, *p*s < 0.001. The interaction reflected a significantly smaller switch cost for the trained participants during the post- than pre-training assessment under the CSI 300 ms, *F*(1, 30) = 26.54, *p* < 0.001, *η*_*p*_^2^ = 0.47, and CSI 600 ms, *F*(1, 30) = 4.30, *p* = 0.047, *η*_*p*_^2^ = 0.13, conditions, but not the CSI 800 ms condition, *p* = 0.18. For the control group, the ANOVA only revealed a main CSI effect, *F*(2, 56) = 9.18, *p* = 0.003, *η*_*p*_^2^ = 0.25; other *F*s < 1.27, reflecting a larger switching cost in the CSI 300 ms condition compared to the CSI 600 ms and 800 ms conditions, *p*s < 0.005, which did not differ, *p* = 0.92.

ANOVA of the mixing cost only revealed a main effect of Session, reflecting an overall shorter RT on the post- compared to pre-training test, and a Group × CSI interaction. The interaction reflected a larger overall mixing cost (pooled over the pre- and post-training sessions) for the control group than the training group on CSI-300 ms trials, *F*(1, 58) = 14.50, *p* < 0.001, *η*_*p*_^2^ = 0.20, but not on any of the other two CSI condition trials, *F*s < 1. However, most importantly, none of the other effects involving the group factor were significant. Thus, training did not result in a larger decrease in mixing cost from pre- to post-training assessment session than that observed in the control group, for any of the CSI conditions.

#### Stroop task

One participant from the training group did not respond correctly on any of the congruent trials of the pre-training assessment session and her data were not used in the analysis. Figure 3 suggests no major decrease in interference score from the pre- to post-training assessment for either group. ANOVA indeed did not reveal any significant main or interaction effects.

#### Flanker task

Figure 3 shows that, in both groups, the difference between incongruent and congruent trials (interference score) remained largely unchanged from pre- to post-training assessment. The Group × Session ANOVA on the interference score indeed did not reveal any significant effects.

#### WM tasks

Figure 3 shows that both groups showed better performance on the easy than the difficult WM task, and better performance on the post- compared to pre-training assessment session for both versions of the WM task. A Group × Task × Session ANOVA only revealed a main effect of Session, reflecting the general improvement from pre- to post-training assessment, and of Task, reflecting better performance on the easy than the difficult task.

#### Go/no-go task

For Go/no-go task performance, expressed as the difference between hits and false alarms, Fig. 3 suggests no large differences between and within groups. This was confirmed by ANOVA, which did not reveal any significant effects. ANOVA using the go-RT data only revealed a significant Group × Session interaction. The trained group displayed shorter RTs during the post-training assessment (*M* = 418.28, SD = 37.61) than during the pre-training assessment (*M* = 437.39, SD = 49.64; *F*(1, 30) = 5.43, *p* = 0.03, *η*_*p*_^2^ = 0.15), whereas the control group displayed the reversed pattern: longer RTs during the post-training session (*M* = 429.19, SD = 42.10) compared to the pre-training session (*M* = 415.23, SD = 39.65; *F*(1, 30) = 5.04, *p* = 0.03, *η*_*p*_^2^ = 0.15).

#### RAPM

Figure 3 shows no large difference in performance on the RAPM as a function of group and/or assessment session. ANOVA did not reveal any significant effects.

## Discussion

The present study examined the effect of extensive task-switching training in young adults. We measured training-induced effects on the trained task itself, on tasks measuring (near) transfer to another switching task, and on tasks measuring far transfer to a variety of executive functions and general intelligence. Across training sessions, there was a reduction of the switch cost for trials with a 300 and 600 ms CSI, which reached an asymptote relatively early in training, but not for trials with the longest, 800 ms, CSI. The mixing cost decreased to the same extent in each of the CSI conditions, also reaching an asymptote relatively early in training. At the end of training, there were still residual switch and mixing costs. In terms of transfer, we only found evidence of a reduction of the switch cost in the CSI 300 and 600 ms conditions of the transfer-switching task, reflecting near transfer. Relative to the performance of the control participants, training did not induce any (additional) beneficial effects for any of the other (far) transfer tasks.

The switch cost reduction across training sessions is in line with previous studies examining other age groups, types of participant (clinical vs. healthy), and/or using other switch tasks for training (Karbach & Kray, 2009; Kray et al., 2012; Minear & Shah, 2008; White & Shah, 2006; Zinke et al., 2012). These studies used a much more limited number of training sessions (ranging from two to four sessions compared to 21 sessions in the present study) involving a lower number of total mixed-task training trials (in the order of maximally 1700 trials for previous studies compared to 4473 trials in the present study). However, our more extensive training resulted in a stronger reduction of switching cost (relative to the first training session) compared to these previous studies. For example, this reduction was maximally 60% in Minear and Shah, 2008 (see Experiment 2, RGXO 200 ms CSI task). As can also be inferred from Fig. 1, in our study the percentage reduction for the CSI 300 ms condition showed a somewhat irregular pattern, reaching a value of 74% on Session 6, followed by a drop to a mean of 59% on Sessions 7–11, reaching a maximum of 81% on Session 13, again followed by a temporary drop to a mean of 58%, and ending up with 73% on the final training sessions. This pattern suggests that it took quite some training to more-or-less stabilize the switch cost reduction at this relatively high level. The fact that the reduction was limited to trials with a relatively short CSI supports the results of the study by Wendt et al. (2017), who used a training task that was similar to our switching transfer task. These results suggest that both our and their type of task-switch training specifically enhances the efficiency of task-set preparation and/or (previous) task-set inhibition processes.

We also assessed training-induced changes in mixing cost. Consistent with previous studies (Minear & Shah, 2008; Soveri et al., 2013; Strobach et al., 2012), we found a considerable reduction in mixing cost across training. Moreover, across all sessions, mixing costs were larger for the shortest CSI compared to the longer CSIs. This general effect is consistent with earlier (non-training) studies finding a smaller mixing cost with a long (1000 ms) compared to a short (100 ms) CSI, possibly reflecting the effect of a better preparation for, and resolution of, the task-set conflict associated with the trials of mixed-task blocks (Lawo et al., 2012; Rubin & Meiran, 2005). However, based on this general CSI effect, one could have expected that training effects, in terms of mixing-cost reduction, would have become especially visible under our shortest CSI conditions, under the assumption that training enhanced the efficiency of processes related to task-set conflict resolution. There might at least be two reasons why we observed a comparable decrease in mixing cost under the three CSI training conditions in our study. First, the mixing cost, and the change thereof across training sessions, might have primarily reflected other processes that have been argued to underlie mixing costs, such as general differences in arousal and working memory load associated with maintaining multiple task sets (Rogers & Monsell, 1995). Arguably, such processes are less sensitive to the CSI. However, if learning across training sessions would reflect a decrease in arousal or working memory load it remains unclear why we did not see a beneficial effect for any of the transfer tasks, specifically the transfer switch and WM tasks. Second, the two extreme CSI values in our study (300 vs. 800 ms) were smaller than those in the corresponding previous studies and might have been insufficiently different to yield reliable mixing-cost differences.

Although very small, switch and mixing costs were still statistically significant at the end of training. These remaining costs are in line with residual switch and mixing costs frequently observed in (non-training) task-switching studies manipulating CSI (see Vandierendonck et al., 2010, for a review): costs do not disappear entirely even after long CSIs. Residual switch costs have been argued to be reconcilable with both the reconfiguration and interference accounts of the switch cost. The reconfiguration account can deal with residual switch costs by assuming the existence of both an endogenous reconfiguration stage (which is sensitive to CSI) and an exogenous stage that is only activated at the time of presentation of the target stimulus and thus not sensitive to CSI (Rogers & Monsell, 1995). Another variant of the reconfiguration account suggests that residual costs reflect occasional failures in, or variations in timing of, preparatory processes across trials (e.g., Brown, Lehmann, & Poboka, 2006). The interference account may assume the operation of interfering events that are independent of the duration of task-set preparation, such as those based on the retrieval of competing stimulus–response associations at the time of presentation of the target stimulus (e.g., Wylie & Allport, 2000). Residual mixing costs in young adults have been found to result from interference at the level of stimulus processing, specifically interference from the irrelevant stimulus feature (Whitson et al., 2014). In any case, whatever the nature of the mechanism underlying the residual costs found in previous studies may be, the present study suggests that even extensive training does not eliminate them.

Task-switch training had beneficial effects on a transfer switch task. Specifically, there was a significant reduction of the switch cost from the pre- to post-training assessment under the CSI 300 and 600 ms conditions for the trained but not control participants. This beneficial transfer effect suggests that training on a task with two-dimensional stimuli does not merely enhance the efficiency of preparatory processes related to biasing one perceptual dimension (see also Wendt et al., 2017). If this were the case, there would be no reason to expect any positive training-induced effects on the transfer task, which involved one-dimensional stimuli (black-colored digits). Moreover, the finding that beneficial transfer effects were limited to the two shortest CSI conditions further supports the claim that training specifically enhanced preparatory rather than retroactive, target-stimulus-induced processes.

We did not observe any beneficial training-induced effects on the mixing cost in the transfer-switching task, which is not in line with a number of previous studies (Karbach & Kray, 2009; Kray & Fehér, 2017; Kray et al., 2012; Minear & Shah, 2008; Zinke et al., 2012). These studies revealed training-induced reductions in transfer-task mixing cost, either with (Karbach & Kray, 2009; Kray et al., 2012) or without (Kray & Fehér, 2017; Minear & Shah, 2008; Zinke et al., 2012) a concomitant training-induced reduction in switch cost. At present, the reason for these between-study differences in near-transfer effects, when differentiating between switch- and mixing-cost benefits, is unclear. The lack of any beneficial far-transfer effects is in line with previous recent task-switching training studies (e.g., Grönholm-Nyman et al., 2017; Kray & Fehér, 2017; Pereg et al., 2013; von Bastian & Oberauer, 2013; Zinke et al., 2012). The lack of any transfer to Stroop task performance is, perhaps, especially worthy of note. Like the trained task, the Stroop task contained bi-dimensional stimuli and involved four trial types, two congruent and two incongruent trials in which the task-irrelevant feature (e.g., switch task: color of letter; Stroop: color word) was consistent and not consistent with the response to the target feature (e.g., switch task: letter; Stroop: color of word), respectively. Hence, training on the task-switching task could be assumed to at least also involve training to inhibit responding to task-irrelevant features in other tasks, like the Stroop task. However, there are a few important differences between the switch and Stroop tasks that might have limited transfer, such as the presence/absence of a preparatory cue (present in the switch but not Stroop task), the nature of the interfering irrelevant feature (which likely implies a stronger interference in case of the Stroop than the switch task), and switching demand (strong for the switch task involving a switch of attention between two stimulus features; absent for the Stroop task implicating continued attention to the same target dimension). In any case, the lack of training-induced benefits for the Stroop task underscores the relative task-specific nature of the processes that were enhanced through training. Although the extent of far-transfer effects may be modulated by variables such as the cognitive demands of the specific training task (see also below) and the age and type of participants, the present null results are in line with the general training literature, mostly consisting of working memory training studies, suggesting no or very limited far-transfer effects (Melby-Lervåg et al., 2016).

### Limitations and future directions

Although we used an extensive training protocol, the difficulty of the task was not adaptive. Task difficulty, either in terms of inter-stimulus interval, stimulus display duration, and/or number of tasks or response alternatives, remained constant and did not vary contingent upon the participant’s performance level. Moreover, using a cued switching task we reduced working memory demands relative to when using an uncued switching task in which the participant has to keep track of the current task. These features may have limited the potential of the training to yield far-transfer effects, for example also by limiting the motivation to perform the training task. However, it is important to note that there were no signs that the participants lost motivation to perform well on the trained task, as is reflected in the continued near-asymptotic high accuracy of responding across the training sessions in combination with the continuous decrease in RTs. Moreover, with a few exceptions (e.g., Anguera et al., 2013; Grönholm-Nyman et al., 2017; von Bastian & Oberauer, 2013) most training studies did not use adaptive training protocols, and overall the (non-)adaptive nature of the task does not seem to be critical to finding or not finding far-transfer effects. The same seems to be true for other measures that were taken in some studies to maintain motivation during training, such as by providing performance-dependent (monetary) feedback (e.g., Dörrenbächer et al., 2014; Strobach et al., 2012): training and/or transfer effects may still be limited despite such measures. Finally, although the WM demands of our (cued switching) training task may have been relatively low, it could be argued that it did involve a more clear (additional) inhibition component relative to tasks employing stimuli for which the different features were presented separately (e.g., a side-by-side presentation of a digit and a letter, e.g., Kray & Frehér, 2017) rather than integrated into one stimulus. As suggested by Kray & Fehér (2017), the latter mode of presentation may implicate a stronger selective attentional or inhibitory demand than the former. However, the present study suggests that this feature too may not be critical for (not) finding far transfer.

A further limitation concerns the battery of tasks used for assessing transfer. Although we used a relatively varied set of instruments, covering the most important aspects of executive functioning, all functions, except for interference control that was covered by both the Stroop and Flanker tasks, were assessed with a single task. This may have enhanced the influence of task-specific aspects and the use of more than one task to cover each component would have been preferred. However, it must be noted that there was not even a hint of a beneficial training-induced effect for any of the far-transfer tasks, although except for perhaps the easy WM task there seemed to be sufficient room for (further) performance improvement from pre- to post-training assessment (no ceiling effects).

The control activity performed by the participants in the control group, making sand paintings, was also used in a previous study (Zhao, Chen, & Maes, 2016). This task was chosen so as to equate the training and control groups on a number of aspects: (1) experience with environmental factors associated with the study, (2) being involved in an engaging task, and (3) believing that one was involved in some treatment program, thereby reducing potential differences in expectancy effects. One potential limitation of this control activity is that it does not control for experience with speeded response computer tasks. Indeed, there were several indications suggesting that, compared to the control participants, the participants from the trained task had learned to become faster in responding in computer tasks in general. Specifically, whereas the control participants displayed longer go-trial RTs on the post- compared to pre-training go/no-go task, the trained participants showed the reverse pattern. In addition, for both the Stroop and Flanker task, Fig. 3 suggests shorter RTs for all trial types for the trained compared to control group at the post-training but not pre-training session, an impression that was statistically confirmed by corresponding analyses (results not shown). However, for the latter tasks it is important to note that the faster responding held for all trial types and that the outcome measure used for each of these tasks (difference score) was not affected by this general RT difference between the two groups. In our previous study using this control condition we suggested that this task also may demand EFs, specifically response inhibition. To the extent that this assumption is true (unfortunately, we do not have any additional data that directly speaks to this issue), this control condition would constitute a rather conservative control condition, limiting the chance of finding differences with the trained condition. However, even when assuming the involvement of inhibitory processes in the control task, at least the transfer-switching task must also have involved other processes that were trained in the training task, given the transfer task-switch benefits (at least in terms of switch costs) that were seen in the trained relative to the control group. The lack of a difference in pre- to post-training improvement between the trained and control groups for the mixing cost in the transfer-switching task and the accuracy data of the WM tasks may, in principle, be due to the activities performed by the trained and control participants accidentally and unintentionally enhancing EFs to a similar extent, leading to an equally enhanced performance on these tasks. However, it may be more likely that this similar improvement reflected a test–retest, practice effect in both groups which might also have implied a floor effect with respect to finding additional beneficial training effects. In any case, future research should ideally also incorporate other appropriate control conditions.

A final limitation is the lack of a follow-up measurement for the training and near-transfer benefits. This limitation is shared with the large majority of task-switching training studies. The few studies that did incorporate a follow-up measurement (e.g., Grönholm-Nyman et al., 2017: 1-year follow-up; Kray & Fehér, 2017: 6-month follow-up) suggest limited maintenance, at least with respect to near-transfer effects, although this may be modulated by age of the participants. However, future research should standardly include follow-up measurements.

## Conclusion

This study assessed training and transfer effects, and the nature of the processes underlying them, as induced by an extensive cued task-switching training program. Training reduced both switch and mixing costs and the asymptote of these reductions was reached relatively early in the training program. Continued training on the task, implicating overtraining, did not fully reduce the costs, as reflected in residual costs at the end of training. The switch cost reduction was restricted to trials with a relatively short CSI, whereas the mixing cost decreased under each of the three CSI conditions. Based on a comparison with an active control group, training benefitted performance on a switching task with other stimuli, reflecting near transfer. However, this benefit was limited to the switch cost and to trials with a relatively short CSI. No evidence was obtained of training-induced beneficial effects on far-transfer tasks measuring interference control, response inhibition, WM, and general IQ. This study suggests that even extensive cued task-switch training does not result in far-transfer effects and that such training specifically enhances the efficiency of processes that are sensitive to the CSI, such as those involved in preparing for the relevant upcoming task set and/or inhibition of the previous task set. Furthermore, the lack of beneficial far-transfer effects supports failures of far-transfer effects in previous task-switch training studies using less extensive training and in cognitive training studies in general.

## Electronic supplementary material

Below is the link to the electronic supplementary material.

Supplementary material 1 (DOCX 277 KB)
