# Supplementary material for: Training and transfer effects of extensive task-switching training in students
Source: Psychol Res. 2018 Jul 17;84(2):389–403. doi: 10.1007/s00426-018-1059-7 (PMC7039846; doi:10.1007/s00426-018-1059-7)
Supplement: Supplementary file 1 — Supplementary material 1 (DOCX 277 KB) [file 426_2018_1059_MOESM1_ESM.docx]

**Performance accuracy for the training and transfer switching tasks, the Stroop task, and the flanker task: data and analyses**

1) Training

*Figure 1*. Top three panels: Mean proportion of trials with a correct response on each of the three trial types (switch, non-switch, and single-task trials) on each of the 21 training sessions. Bottom three panels: Mean switch and mixing costs across the training sessions. Results are depicted separately for each of the three CSI conditions (30, 600, and 800 ms).

1a) Results of CSI × Session ANOVA on switch cost (accuracy non-switch trials minus accuracy switch trials), with Greenhouse-Geisser correction:

CSI: *F*(2, 60) = 1.68, *p* = .20, *η_p_*² = .05

Session: *F*(20, 600) = 1.25, *p* = .26, *η_p_*² = .04

CSI × Session, *F*(40, 1200) = 0.95, *p* =.51, *η_p_*² = .03

1b) Results of CSI × Session ANOVA on mixing cost (accuracy single-task trials minus accuracy non-switch trials), with Greenhouse-Geisser correction:

CSI: *F*(2, 60) = 11.34, ***p* < .001**, *η_p_*² = .27

Session: *F*(20, 600) = 1.01, *p* = .44, *η_p_*² = .03

CSI × Session, *F*(40, 1200) = 1.08, *p* =.34, *η_p_*² = .04

Main effect of CSI reflects lower difference score for CSI 300 (-0.005) than CSI 600 (0.005), *p* < .001. There was no difference between the CSI 300 and CSI 800 (-0.001) conditions, *p* = .10.

2) Transfer switch task

*Figure 2*. Groups’ mean (+SEM) proportion of trials with a correct response on the three different trial types (switch, non-switch, and single-task trials) of the transfer switching task, separately for pre- and post-training assessment session and CSI condition.

2a) Results of Group × CSI × Session ANOVA on switch cost (accuracy non-switch trials minus accuracy switch trials), with Greenhouse-Geisser correction:

Group: *F*(1, 58) = 5.49, ***p* = .02**, *η_p_*² = .09 🡺 Training group (.028) > Control group (.011)

CSI: *F*(2, 116) = 2.49, *p* = .09, *η_p_*² = .04

Session: *F*(1, 58) = 7.02, ***p* = .01**, *η_p_*² = .11 🡺 Session 1 (.011) < Session 2 (.017)

Group × Session: *F*(1, 58) = 1.03, *p* = .31, *η_p_*² = .02

Group × CSI: *F*(2, 116) = 2.66, *p* = .08, *η_p_*² = .04

CSI × Session: *F*(2, 116) = 3.57, ***p* = .03**, *η_p_*² = .06

Group × CSI × Session: *F*(2, 116) = 3.20, ***p* = .048**, *η_p_*² = .05 🡺 CSI × Session interaction for

Trained group, reflecting *larger* switch cost on post- than pre-training in 800-ms CSI condition, *p* = .004, but not in other CSI conditions, *p*s > .07. No CSI × Session interaction for control group, *p* = .053.

2b) Results of Group × CSI × Session ANOVA on mixing cost (accuracy single-task trials minus accuracy non-switch trials), with Greenhouse-Geisser correction:

Group: *F*(1, 58) = 3.08, *p* = .09, *η_p_*² = .05

CSI: *F*(2, 116) = 0.51, *p* = .60, *η_p_*² = .01

Session: *F*(1, 58) = 1.62, *p* = .21, *η_p_*² = .03

Group × Session: *F*(1, 58) = 3.38, *p* = .07, *η_p_*² = .06

Group × CSI: *F*(2, 116) = 0.32, *p* = .72, *η_p_*² = .01

CSI × Session: *F*(2, 116) = 4.49, ***p* = .01**, *η_p_*² = .07 🡺 only effect of Session (decrease in

mixing cost) in 800-ms CSI condition, *p* = .02, other *p*s > .33.

Group × CSI × Session: *F*(2, 116) = 2.15, *p* = .12, *η_p_*² = .04

3) Stroop task

*Figure 3*. Groups’ mean (+SEM) proportion of correct responses for incongruent, congruent, and neutral trials of the Stroop task, separately for pre- and post-training sessions.

3a) Results of Group × Session ANOVA on the interference score (accuracy on congruent trials minus accuracy on incongruent trials):

Group: *F*(1, 57) = 0.11, *p* = .74, *η_p_*² = .00

Session: *F*(1, 57) = 2.90, *p* = .09, *η_p_*² = .05

Group × Session: *F*(1, 57) = 0.00, *p* = .98, *η_p_*² = .00

4) Flanker task

4a) Results of Group × Session ANOVA on the interference score (accuracy on congruent trials minus accuracy on incongruent trials):

Group: *F*(1, 57) = 0.63, *p* = .43, *η_p_*² = .01

Session: *F*(1, 57) = 6.92, ***p* = .01**, *η_p_*² = .11 🡺 pre (.00) < post (.02)

Group × Session: *F*(1, 57) = 0.29, *p* = .60, *η_p_*² = .01
